# Age at diagnosis in patients with chronic congenital endocrine conditions: a regional cohort study from a reference center for rare diseases

**DOI:** 10.1186/s13023-021-02099-3

**Published:** 2021-11-04

**Authors:** Wafa Kallali, Claude Messiaen, Roumaisah Saïdi, Soucounda Lessim, Magali Viaud, Jerome Dulon, Mariana Nedelcu, Dinane Samara, Muriel Houang, Bruno Donadille, Carine Courtillot, GianPaolo de Filippo, Jean-Claude Carel, Sophie Christin-Maitre, Philippe Touraine, Irene Netchine, Michel Polak, Juliane Léger

**Affiliations:** 1 Pediatric Endocrinology-Diabetology Department, Reference Center for Growth and Development Endocrine Diseases, Robert Debré University Hospital, Assistance Publique-Hôpitaux de Paris, Université de Paris, 48 Bd Sérurier, 75019 Paris, France; 2 Banque Nationale de Données Maladies Rares, DSI-I&D, APHP, Paris, France; 3 Pediatric Endocrinology, Gynecology and Diabetology Department, Reference Center for Growth and Development Endocrine Diseases, Necker University Hospital, Assistance Publique-Hôpitaux de Paris, Université de Paris, 75015 Paris, France; 4 Pediatric Endocrinology Unit, Reference Center for Growth and Development Endocrine Diseases, Trousseau University Hospital, Assistance Publique-Hôpitaux de Paris, Sorbonne Université, 75012 Paris, France; 5 Endocrinology Department, Reference Center for Growth and Development Endocrine Diseases, Saint Antoine University Hospital, Assistance Publique-Hôpitaux de Paris, Sorbonne Université, 75012 Paris, France; 6 Endocrinology Department, Reference Center for Growth and Development Endocrine Diseases, La Pitié Salpétrière University Hospital, Assistance Publique-Hôpitaux de Paris, Sorbonne Université, 75013 Paris, France

**Keywords:** Diagnosis, Congenital endocrine disease, Cohort, Female, Male

## Abstract

**Background:**

For chronic congenital endocrine conditions, age at diagnosis is a key issue with implications for optimal management and psychological concerns. These conditions are associated with an increase in the risk of comorbid conditions, particularly as  it concerns growth, pubertal development and fertility potential. Clinical presentation and severity depend on the disorder and the patient’s age, but diagnosis is often late.

**Objective:**

To evaluate age at diagnosis for the most frequent congenital endocrine diseases affecting growth and/or development.

**Patients and Methods:**

This observational cohort study included all patients (*n* = 4379) with well-defined chronic congenital endocrine diseases—non-acquired isolated growth hormone deficiency (IGHD), isolated congenital hypogonadotropic hypogonadism (ICHH), ectopic neurohypophysis (NH), Turner syndrome (TS), McCune-Albright syndrome (MAS), complete androgen insensitivity syndrome (CAIS) and gonadal dysgenesis (GD)—included in the database of a single multisite reference center for rare endocrine growth and developmental disorders, over a period of 14 years. Patients with congenital hypothyroidism and adrenal hyperplasia were excluded as they are generally identified during neonatal screening.

**Results:**

Median age at diagnosis depended on the disease: first year of life for GD, before the age of five years for ectopic NH and MAS, 8–10 years for IGHD, TS (11% diagnosed antenatally) and CAIS and 17.4 years for ICHH. One third of the patients were diagnosed before the age of five years. Diagnosis occurred in adulthood in 22% of cases for CAIS, 11.6% for TS, 8.8% for GD, 0.8% for ectopic NH, and 0.4% for IGHD. A male predominance (2/3) was observed for IGHD, ectopic NH, ICHH and GD.

**Conclusion:**

The early recognition of growth/developmental failure during childhood is essential, to reduce time-to-diagnosis and improve outcomes.

## Introduction

For chronic congenital endocrine conditions, age at diagnosis is a key issue with implications for optimal management and psychological concerns. These conditions are associated with a high risk of comorbid conditions, affecting growth, pubertal development and fertility potential, in particular. Clinical presentation and severity depend on the disorder and the patient’s age, but diagnosis is often late. There are several registries for endocrine disorders, but age at the detection of chronic congenital conditions has never been studied systematically [1]. Reports have been published for single centers and single conditions, but no information is available for large cohorts of patients with rare chronic congenital endocrine disorders. Early diagnosis and treatment have been shown to have a positive impact on the outcome of patients with congenital hypothyroidism and congenital adrenal hyperplasia. Most patients with these conditions are now diagnosed within the first three weeks of life, thanks to the introduction of neonatal screening programs [2, 3].

Reference centers for rare diseases were established in France under the Rare Disease Plan, which began in 2004 [4]. Their principal missions are to improve care and healthcare equality for all patients with rare conditions, and to decrease the time-to-diagnosis and the number of cases of undiagnosed disease. They have helped to improve our knowledge and expertise in the study and treatment of rare diseases, with potential implications for improving outcome. A large national database (the *Cemara database*) including cases of rare diseases from all the reference centers in France and using standardized data collection methods has been established, with the aim of improving our understanding of the epidemiology of rare diseases and promoting research activities [5].

The aim of this study was to evaluate age at diagnosis in rare congenital endocrine diseases affecting growth and/or development, based on the database of our reference center.

## Patients and methods

### Patients

This observational cohort study included all patients (*n* = 4379) diagnosed with seven well-defined chronic congenital endocrine diseases included in the database of a single reference center for rare endocrine growth and developmental disorders. This multisite reference center includes five academic pediatric and adult endocrinology departments in the Paris area, and the data considered covered a period of 14 years, between January 2006 (when this group of departments qualified as a reference center for rare diseases) and December 2019. Patients with congenital hypothyroidism and adrenal hyperplasia were excluded, as these conditions are generally diagnosed during neonatal screening.

Patients were considered eligible for the study if they were included in the database of our reference center during this period and had non-acquired idiopathic and isolated growth hormone deficiency (IGHD), ectopic neurohypophysis (ectopic NH), isolated congenital hypogonadotropic hypogonadism (ICHH), Turner syndrome (TS), McCune-Albright syndrome (MAS), or disorders of genital development, such as complete androgen insensitivity syndrome (CAIS) and gonadal dysgenesis 45X, 46XY (GD).

### Study protocol

We analyzed this reference center database, which includes clinical information extracted from the medical records of all patients with these rare congenital endocrine diseases. The demographic characteristics recorded included date of birth, sex, age and year at diagnosis.

The study protocol was approved by the Paris Nord Ethics Review Committee for Biomedical Research Projects (CEERB) (No. 12-029). All the patients’ files were included in the CEMARA database. This national database has been declared to the French data protection agency (*Commission Nationale de l’Informatique et des Libertés* (CNIL), No. 909474 in 2010). In compliance with French law, each of the patients included expressed their non-opposition to the collection and use of personal data.

### Methods

The total number of patients was recorded for each condition and each year during the study period. We recorded the number of new patients and the number of patients with long-term care. New patients were defined as any patient not previously seen by the healthcare provider. Patients were classified into two groups, with seven subgroups, on the basis of their chronic pathological endocrine conditions: (1) pituitary insufficiency, with the subgroups non-acquired idiopathic isolated growth hormone deficiency, ectopic neurohypophysis and isolated congenital hypogonadotropic hypogonadism; (2) syndromic or gonadal disorders, with the subgroups Turner syndrome, McCune-Albright syndrome, complete androgen insensitivity syndrome and gonadal dysgenesis.

Each rare disease had specific features based on clinical, hormonal, cytogenetic, molecular and imaging evaluations. Patients with IGHD had growth failure caused by isolated GH deficiency, with no identifiable etiology and no structural hypothalamic pituitary abnormalities on magnetic resonance imaging (MRI). Biochemical markers included low serum IGF-I levels and GH peaks < 6.7 ng/ml in two independent GH stimulation tests. Patients with ectopic NH were diagnosed mostly on the basis of hypoglycemia, midline abnormalities and/or short stature and, in addition, for male patients, micropenis and/or cryptorchidism. In all cases, MRI findings showed an ectopic location of the posterior lobe of the pituitary gland, with low serum IGF-I levels and, in most cases, a GH peak < 6.7 ng/ml in at least one GH stimulation test. Patients with ICHH were identified either during infancy, on the basis of cryptorchidism, micropenis, familial or syndromic presentation, or later in life, on the basis of an absence of sexual maturation and/or infertility, with a biochemical profile of low concentrations of sex steroids in a context of gonadotropin deficiency. Patients with TS were defined on the basis of their karyotype, with a monosomic or other chromosomal aberration for the X chromosome, determined before or after birth, in female individuals mostly with unexplained growth failure or delayed puberty, cardiac or morphological abnormalities or infertility. Patients with MAS, arising from somatic gain-of function mutations of the *GNAS* gene leading to mosaic Gsα activation, were identified clinically, on the basis of a variable combination of fibrous bone dysplasia, café-au-lait skin pigmentation, and hyperfunctioning endocrinopathies, mostly peripheral precocious puberty in early childhood. Patients with CAIS have normal female external genitalia with a 46, XY karyotype and undescended testes, due to a complete absence of response to androgens. Patients with GD were defined on the basis of a 45, X/46, XY karyotype, with abnormal genitalia.

Patients were classified into age groups: antenatal, [0–1), [1–2), [2–5), [5–8), [8–11), [11–18), [18–25), [25–40), [40–60) and ≥ 60 years, and into group on the basis of age at diagnosis: ≤5 years, [5–11) years, [11–18) years and ≥ 18 years.

Two periods, from 2006 to 2012, and from 2013 to 2019, were considered, for an analysis of trends in diagnosis over time. The influence of sex on age at diagnosis was also studied for patients with IGHD, ectopic NH, ICHH and GD.

### Statistical analysis

The results are expressed as numerical values (percentages) for categorical variables and as medians (interquartile range) for continuous variables. The Wilcoxon test was used for comparisons by sex. The chi-squared test was used to assess temporal trends in age at diagnosis between the two time periods considered. A *p*-value less than 0.05 was considered statistically significant. Data were analyzed with R software version 4.0.2.

## Results

**Fig. 1:**
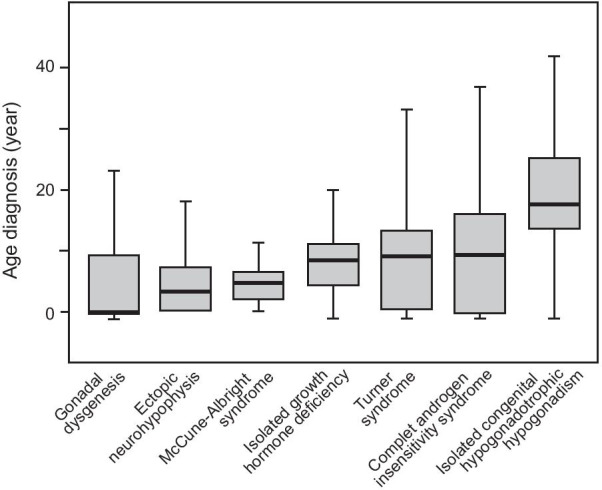
Median age at diagnosis in patients with gonadal dysgenesis (*n* = 68), ectopic neurohypophysis (*n* = 364), McCune-Albright syndrome (*n* = 38), isolated and idiopathic GH deficiency (*n* = 1593), Turner syndrome (*n* = 1478), complete androgen insensitivity syndrome (*n* = 77), and isolated congenital hypogonadotropic hypogonadism (*n* = 761). The horizontal line represents the median, the box indicates the interquartile range (50% of values), and the whiskers show the range of the data, excluding the outliers. ND: not determined

**Fig. 2:**
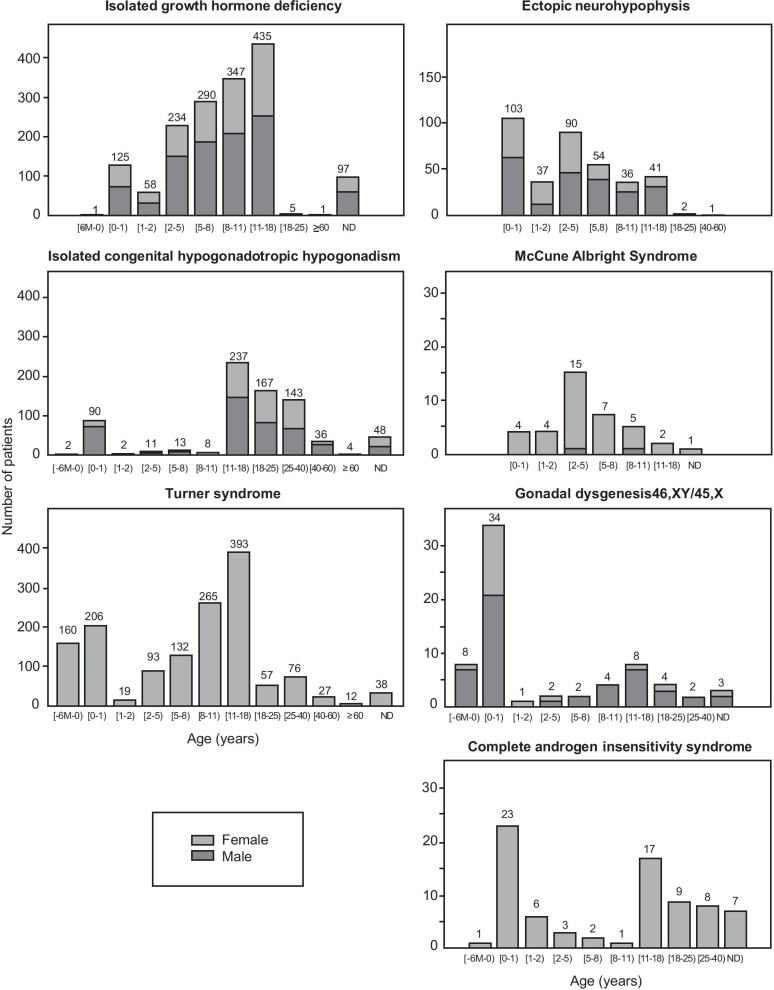
Age and sex distribution at diagnosis in patients with seven chronic congenital endocrine diseases affecting growth/development: isolated and idiopathic GH deficiency (*n* = 1593), ectopic neurohypophysis (*n* = 364), isolated congenital hypogonadotropic hypogonadism (*n* = 761), McCune-Albright syndrome (*n* = 38), complete androgen insensitivity syndrome (*n* = 77), gonadal dysgenesis (*n* = 68), and Turner syndrome (*n* = 1478).

The median (interquartile range) age at diagnosis, and the distribution of age at diagnosis by congenital endocrine disease, age group and sex are shown in Figs. 1, 2 and Table 1.

**Table 1:** Age at diagnosis for seven congenital endocrine diseases, by age category

	Number of patients (Female%)	Missing data *N* *(%)*	Median age at diagnosis (years)	Patients diagnosed < 5 years old	Patients diagnosed [5–11) years old	Patients diagnosed [11–18) years old	Patients diagnosed ≥18 years old
Idiopathic isolated growth hormone deficiency	1593 *(38.5%)*	97 *(6.1%)*	8.42 (4.42-11.33)	418 *(26.2%)*	637 *(40%)*	435 *(27.3%)*	6 *(0.4%)*
Ectopic posterior pituitary syndrome	364 *(38.7%)*	0	3.42 (0.50-7.50)	230 *(63.2%)*	90 *(24.7%)*	41 *(11.3%)*	3 *(0.8%)*
Isolated congenital hypogonadotropic hypogonadism	761 (*39.3%)*	48 *(6.3%)*	17.42 (14.00-25.00)	105 *(13.8%)*	21 *(2.8%)*	237 *(31.1%)*	350 *(46%)*
Turner syndrome*	1478 *(100%)*	38 *(2.6%)*	9.00** (0.67-13.69)	478 *(32.3%)*	397 *(26.9%)*	393 *(26.6%)*	172 *(11.6%)*
McCune-Albright Syndrome	38 *(94.7%)*	1 *(2.6%)*	4.67 (2.50-6.58)	23 *(60.5%)*	12 *(31.6%)*	2 *(5.3%)*	ND
Complete androgen insensitivity syndrome	77 *(100%)*	7 *(9.1%)*	9.25 (0.21-16.00)	33 *(42.8%)*	3 *(3.9%)*	17 *(22.1%)*	17 *(22.1%)*
Gonadal dysgenesis 46,XY/45,X	68 *(27.9%)*	3 *(4.4%)*	0.00 (0.00-9.25)	45 *(66.2%)*	6 *(8.8%)*	8 *(11.8%)*	6 *(8.8%)*

Values are expressed as median ± quartiles, or number *(%)*

*Antenatal diagnosis in Turner syndrome: *n* = 160 (10.8%)

**Median age of patients with Turner syndrome diagnosed postnatally: 10.83 (7.00–15.00) years

IGHD (mostly followed exclusively at pediatric endocrine centers) and TS were the most frequent congenital endocrine conditions in our cohort, accounting for 70.1% (*n* = 3071) of the patients. ICHH and ectopic NH accounted for 17.4% (*n* = 761) and 8.3% (*n* = 364), respectively. MAS, CAIS and GD were very rare, each accounting for less than 2% of our cohort.

Due to its effects on genital development, GD was mostly diagnosed antenatally or before the age of one year. More than 60% of the patients with ectopic NH and MAS were diagnosed before the age of five years. Median age at diagnosis was 8.4 years for IGHD, 9.0 years for TS, 9.3 years for CAIS and 17.4 years for ICHH, which was mostly diagnosed during adolescence (31.1% of cases) or in adults (46%). Unexpectedly, diagnosis occurred late, during adulthood, in a few patients with other conditions, such as GD in 8.8% of cases, ectopic NH in 0.8%, and IGHD in 0.4% of cases, and for conditions involving amenorrhea and infertility, such as TS and CAIS, in 11.6% and 22.1% of cases, respectively. Interestingly, 10.8% of patients with Turner syndrome were diagnosed antenatally.

The sex ratio differed between endocrine diseases, with a male predominance for IGHD (61.5%), ectopic NH (61.3%), ICHH (60.7%) and GD (72.1%). As expected, a female predominance was observed in MAS (94.7%) and, by definition, all the patients with TS and CAIS were considered female. Age at diagnosis was determined by sex for IGHD, ectopic NH, ICHH and GD (Table 2). Despite an absence of difference between the sexes for each disease *per se*, male predominance was observed in almost all age groups for IGHD and ICHH (Fig. 2). For patients with ectopic NH, a balanced sex ratio was observed for patients under the age of 5 years, but male predominance was observed for older patients (Fig. 2). The effect of sex on median age at diagnosis also differed between endocrine disorders, with earlier diagnosis for female patients than for male patients for ectopic NH, and for male patients than for female patients for IGHD {2.50 (0.83–4.83) vs. 4.00 (0.50–8.27) years, *p* = 0.001} and {8.83 (4.33–11.25) vs. 8.17 (4.50–11.42) years, *p* = 0.07; for female and male patients, respectively}.

**Table 2:** Age at diagnosis, by sex, in patients with isolated growth hormone deficiency, ectopic neurohypophysis, isolated congenital hypogonadotropic hypogonadism and gonadal dysgenesis

	Female Median age (interquartile range), in years	Male Median age (interquartile range), in years	*p*
Isolated growth hormone deficiency	8.83 (4.33–11.25) *n* = 614	8.17 (4.50–11.42) *n* = 979	0.073
Ectopic posterior pituitary syndrome	2.50 (0.83–4.83) *n* = 141	4.00 (0.50–8.27) *n* = 223	0.001
Isolated congenital hypogonadotropic hypogonadism	18.50 (16.00–26.00) *n* = 299	16.00 (11.88–23.00) *n* = 462	< 0.001
Gonadal dysgenesis 46,XY/45,X	0.00 (0.00–0.08) *n* = 19	0.00 (0.00–11.6) *n* = 49	0.026

Values are expressed as median (interquartile range), or number

No temporal trend in age at diagnosis was observed in comparisons between the 2006–2012 period and the 2013–2019 period, for any of the conditions studied (data not shown).

## Discussion

We report the largest regional multicenter cohort study to date evaluating the epidemiology of congenital endocrine conditions followed at academic pediatric and adult endocrinology centers. This study demonstrates differences in age at diagnosis for seven congenital chronic endocrine diseases, according to the underlying diagnosis and sex. Most patients with these congenital endocrine disorders were diagnosed during the pediatric period, but large differences were found between groups and between patients within the same etiological group.

Age at diagnosis has been analyzed separately in some chronic congenital conditions. For the most frequently studied conditions, such as IGHD, ectopic NH and TS, considerable attention has been paid to age at the start of GH treatment, which is generally considered to be close to age at diagnosis [6, 7]. In our study, the median age at IGHD diagnosis was similar to the values previously reported, which range from 6.1 to 10.8 years [8–11]. For patients with ectopic NH, diagnosis occurred earlier, as reported in most studies, probably reflecting the more severe phenotype of this condition relative to IGHD in terms of the severity of growth hormone deficiency, resulting, in most cases, in a greater height deficit with respect to mid-parental target height, hormonal deficits affecting two or more additional pituitary hormones, and clinical symptoms early in infancy, including hypoglycemia, prolonged jaundice and/or micropenis and/or cryptorchidism in male patients and/or associated extrapituitary midline malformations leading to cerebral MRI examinations revealing developmental disorders of the hypothalamic pituitary axis [12–15]. Our estimate that more than one quarter of our patients with ectopic NH were diagnosed during the first year of life is consistent with this greater severity.

Another interesting finding for IGHD and ectopic NH was the male predominance observed in both these subgroups, consistent with previous findings [6, 7, 9, 10, 13, 15, 16]. However, female patients with ectopic NH were diagnosed earlier than male patients with this condition, suggesting that the disorder was at least as severe in girls as in boys [13, 15]. The observed male predominance is therefore probably less likely to be due to symptoms or greater growth impairment during the pediatric period, and more likely to be due to referral bias, greater concern about short stature in boys and/or more frequent prescription of GH treatment [10, 17].

Late diagnosis was clearly confirmed for patients with TS, relative to previous reports in which median age at diagnosis ranged from 6.6 to 15 years [6, 7, 18–24]. We found that 10.8% of TS patients were diagnosed antenatally, reflecting recent increases in prenatal diagnosis relative to previous reports [25, 26]. Conversely, a failure to diagnose TS early in life, as in pediatric patients diagnosed after the age of 11 years (26.6%) and in patients diagnosed as adults (11.6%), may be attributed to a lack of concern about short stature and/or pubertal delay in girls, depriving patients of appropriate management and potentially leading to greater morbidity and mortality, a lower quality of life and psychosocial deficits [27–29]. Increasing awareness, to favor the earlier diagnosis of Turner syndrome, remains a matter of high priority, not only in girls with a short stature, but also in those with a mild phenotype, delayed puberty or secondary amenorrhea [30]. Clinical expertise and algorithms for optimizing the early detection of abnormal growth in children, thereby reducing time-to-diagnosis for TS and GH deficiency, should be developed to improve the health and outcomes of these patients [31, 32].

As expected, most patients with isolated congenital hypogonadotropic hypogonadism were diagnosed during adolescence or early childhood, as this disorder is characterized by an absence of puberty and by infertility [33]. Unlike previous reports, boys in our cohort were diagnosed significantly earlier than girls [34]. About 12% of patients with this condition, mostly boys, were diagnosed within the first year of life on the basis of other developmental abnormalities, cryptorchidism, micropenis or a familial form of the disorder.

For other rare and complex diseases, such as gonadal dysgenesis and CAIS with differences of sex development (DSD) due to atypical genital phenotypes with respect to genotype, diagnosis often occurred in early infancy. However, more than half the patients with CAIS and one third of those with gonadal dysgenesis were diagnosed after the age of five years, with some not diagnosed until adulthood, due to the considerable variability of clinical presentations related to specific aspects of these disorders [35–38].

Finally, although MAS is equally frequent in girls and boys, a female preponderance was observed in our cohort of patients followed in endocrinology departments, consistent with the findings of previous studies [39, 40]. Gonadal involvement is much more frequent in girls than in boys, with peripheral precocious puberty and metrorrhagia due to estrogen secretion from ovarian cysts occurring in early childhood. Furthermore, diagnosis may be difficult in boys, in whom fibrous bone dysplasia in isolation or in combination with additional features of MAS may be the key to diagnosis. For these reasons, this condition is typically recognized mostly in girls during early childhood. The management of boys by orthopedists/surgeons rather than endocrinologists may account for the female preponderance found in our endocrinology departments.

One of the major strengths of this study is its inclusion of all the patients from a defined population from a reference center for rare diseases including pediatric and adult academic clinical departments for whom age at diagnosis and etiology were known, over a 14-year period. It was, therefore, possible to evaluate subgroups of individuals with different disorders, with a large number of patients in each group. The method used to identify diagnoses was sufficiently sensitive in most cases. However, this study also had several inherent limitations, due to its observational nature and the heterogeneity of study subjects, who were diagnosed at various ages, from the neonatal period into adulthood. Unfortunately, with the method used, we were able to obtain information only about age at diagnosis and sex. No information was available concerning the clinical symptoms leading to the diagnosis of each disorder. This study cannot, therefore, provide further insight into the mechanisms underlying late diagnosis in some patients, particularly for patients diagnosed in adulthood. It was also impossible to control for potential familial cases within this cohort, which might have affected age at diagnosis due to the potentially greater concern of the parents. We attempted to minimize selection bias by assessing a broad population of patients, but some selection bias may have remained and may have affected the accuracy of median age at diagnosis estimates, as patients not followed at endocrinology centers were not evaluated. We cannot, therefore, rule out the possibility that there was a residual selection bias due to the non-inclusion of patients not followed at endocrinology centers, particularly for boys with MAS, who are mostly followed for non-endocrine manifestations of the disease. Finally, our study includes patients recruited from academic departments in a single region of France. This may also have affected the results, due to the omission of affected individuals followed in decentralized healthcare structures. Moreover, given that our institution is a tertiary referral center, there may be a selection bias for patients at higher risk, and it was unknown whether the patients had been seen at another institution before evaluation at our center.

## Conclusion

This study provides further insight into age at diagnosis and its relationship to sex for several major congenital endocrine disorders. These original findings have important clinical implications for patient management, as they highlight the need for the early recognition of features associated with chronic endocrine disorders of childhood onset, potentially improving their long-term outcomes. The potential sex bias in some of these conditions requires further exploration, but our findings highlight the utility of developing algorithms for facilitating the identification of patients at an early age, due to the inherently high risk of these patients developing comorbid conditions. Future studies should also explore the value of these algorithms for application in the primary care setting and improvements in healthcare organization to reduce the time-to-diagnosis for heterogeneous groups of patients with rare conditions. Continuous monitoring of standardized data collection in databases such as the one used here is required, to follow trends and to facilitate large-scale multicenter studies.

## Data Availability

The datasets used and/or analyzed during the current study are available from the corresponding author on reasonable request.

## Abbreviations

IGHD: Isolated growth hormone deficiency
Ectopic NH: Ectopic neurohypophysis
ICHH: Isolated congenital hypogonadotropic hypogonadism
TS: Turner syndrome
MAS: McCune–Albright syndrome
CAIS: Complete androgen insensitivity syndrome
GD: Gonadal dysgenesis
